# Regional Variation in Healthcare Use Among Medicare Beneficiaries With Alzheimer’s Disease and Related Dementias

**DOI:** 10.1093/geroni/igaa057.069

**Published:** 2020-12-16

**Authors:** Elizabeth White, Momotazur Rahman

**Affiliations:** Brown University School of Public Health, Providence, Rhode Island, United States

## Abstract

In this national prospective study we describe regional variation in healthcare utilization among Medicare beneficiaries with Alzheimer’s disease and related dementias (ADRD) in the six years after diagnosis. We use 2008-2015 Medicare claims and other administrative data to map nursing home, home health, hospital, and hospice use across hospital referral regions; and examine the relationship of state and county supply-side factors to time beneficiaries spend in different settings. The sample includes 1,158,655 Medicare fee-for-service beneficiaries diagnosed with ADRD in 2008 and 2009. Nationally, beneficiaries spent a mean of 70.6% of survived days in the community, 23.9% of days in nursing home, and 5.4% of days in hospital. 37.2% of beneficiaries who died within six years had received hospice. Distinct regional patterns emerged. Adjusting for beneficiary and local characteristics, beneficiaries in Midwestern states spent the most time in nursing homes, while beneficiaries in Western states spent the most time in community. The probability of receiving hospice was generally highest in Western and Southern states, and lowest in the Midwest and Northeast. Controlling for beneficiary, local, and state characteristics, we found the following factors to be associated with beneficiaries spending less time in nursing homes: fewer nursing home beds in the county, higher state Medicaid long-term care spending for home and community-based services (HCBS), and state use of Certificate of Need laws. These findings illustrate that state investment in HCBS, and state and local regulation of provider supply are important factors influencing where individuals with ADRD receive care.

